# Defragmenting Mangrove Law Towards Coherent Global Governance

**DOI:** 10.1002/ece3.73721

**Published:** 2026-05-27

**Authors:** Marie Lorber, Paolo Cappa

**Affiliations:** ^1^ Independent Researcher Blagnac France; ^2^ National Marine Science Centre Southern Cross University Coffs Harbour New South Wales Australia

**Keywords:** blue carbon sinks, coastal wetlands, ecosystem services, legal framework, UNCLOS

## Abstract

Mangrove forests are multifunctional coastal ecosystems that support biodiversity and a variety of ecosystem services, including fisheries productivity, coastal stability, climate change mitigation and pollution control. Despite growing scientific awareness of these benefits, mangrove loss continues, mainly due to urban development and the expansion of aquaculture. International governance of mangroves remains fragmented across four major international frameworks, each addressing a distinct dimension of mangrove value though none of them were specifically designed to govern these ecosystems. Mangrove conservation and restoration are addressed through partially overlapping but often uncoordinated objectives, leading to implementation that rarely reflects mangroves as integrated land‐sea systems and resulting in inconsistent and inadequate protection. Rather than promoting a new international treaty, this viewpoint gives a brief legal analysis of international mangrove governance and argues that stronger protection can be achieved through a practical defragmentation strategy based on integrated interpretation and coordinated implementation of existing frameworks. United Nations Convention on the Law of the Sea presents a strong potential with the binding obligation it imposes to protect and preserve the marine environment. The Convention on Biological Diversity focuses on the conservation of biodiversity, but relies on implementation at a national level. The Ramsar Convention, which provides the most explicit international legal bases for mangrove protection, is limited by its largely non‐prescriptive approach. The United Nations Framework Convention on Climate Change offers an interesting framework for the conservation of mangrove ecosystems as carbon sinks, but the lack of coordination with the other instruments means that little consideration is otherwise given to mangroves. An integrated interpretation and coordinated implementation of these four instruments, supported by stronger institutional cooperation, could help translate these distinct mandates into coherent and effective mangrove governance. Finally, to ensure lasting results, this governance must incorporate scientific considerations, including preventing further loss of mangrove forests, avoiding inappropriate restoration, maintaining connectivity between river basins and coasts, anticipating climate‐induced habitat changes and resolving financial and capacity limitations.

## Graphical abstract

Mangrove conservation outcomes are increasingly shaped not only by scientific evidence, but also by how laws and policies translate ecological knowledge into protection measures and restoration practices. Our paper links legal fragmentation to ecological consequences. We summarise the main international frameworks relating to mangroves based on treaty texts, identify gaps created by cross‐sectoral fragmentation and propose practical recommendations to make governance more coherent and ecologically effective, with the aim of allowing concrete improvements in mangrove conservation and restoration.

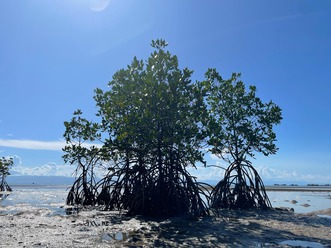

## 1 Introduction

Mangroves comprise a group of approximately 70 known species adapted to the intertidal zone and include various forms such as trees, shrubs, ferns and palms (Romañach et al. 2018; Duke et al. 1998). Global mangrove forests currently cover an area of approximately 147,359 km^2^, mainly distributed in tropical and subtropical regions (Bunting et al. 2022; 2018). Mangroves provide numerous ecosystem services that support both biodiversity and human well‐being (Bimrah et al. 2022). Mangrove forests contribute to coastal protection and geomorphological stability by attenuating wave energy and reducing the impact of storms during tropical cyclones, thus helping to limit coastal damage and erosion (Krauss and Osland 2019). They are also important biodiversity hotspots, providing habitat for numerous terrestrial and aquatic species (Luther and Greenberg 2009) and serving as critical nursery areas for many species of fish and invertebrates (Zu Ermgassen et al. 2025). Furthermore, mangroves play a substantial role in climate regulation through long‐term carbon storage and sequestration, acting as carbon sinks of global importance (Zhu and Yan 2022). More recently, mangrove forests have been recognised for their ability to trap and retain plastic debris, reducing the transport of waste into the open ocean (Cappa et al. 2023; Martin et al. 2019). In addition to their ecological and regulatory functions, mangroves also provide cultural (e.g., tourism, educational) and livelihood value to many coastal communities (Moore et al. 2022). Reflecting these multiple roles, mangrove research has rapidly expanded in recent years, with increasingly interdisciplinary efforts spanning ecology, climate science, coastal protection, socioeconomics and restoration practice (Beddu et al. 2025; Lovelock et al. 2024; Rodríguez‐Rodríguez et al. 2024).

Despite growing scientific awareness of mangrove ecosystems and the wide range of ecosystem services they provide, mangroves are undergoing severe and ongoing decline (Ju et al. 2025; Mcowen et al. 2023). From 1996 to 2020 the world's mangroves experienced a loss of over 5200 km^2^, representing a reduction of 3.4% over 24 years (Bunting et al. 2022). Future projections warn that 25% of mangrove forests are set to be submerged in the next 50 years due to sea‐level rise (IUCN 2024). In many regions, large areas of mangrove forests are being cleared for aquaculture, agriculture and urban development, exposing coastlines to more intense weather events and creating well‐documented risks for local communities (IUNC 2024; van Bochove et al. 2014). The need for a strong and effective framework in mangrove protection is real and urgent. Although multiple legal and policy frameworks for mangrove protection have been established, these remain fragmented at the global level (Slobodian et al. 2025; Walker et al. 2022; Mursyid et al. 2021; Bell‐James et al. 2020; Slobodian and Badoz 2019; Rotich et al. 2016).

Existing international instruments relevant to mangroves—e.g., the United Nations Convention on the Law of the Sea (UNCLOS), the Convention on Biological Diversity (CBD), the Ramsar Convention on Wetlands (Ramsar), the United Nations Framework Convention on Climate Change (UNFCCC)—often overlap and operate in an uncoordinated manner, resulting in insufficient and uneven protection that does not consistently reflect scientific evidence or conservation priorities, leaving key ecological processes and major threats insufficiently addressed (Slobodian et al. 2025; CBD 1992; UNFCCC 1992; UNCLOS 1982; Ramsar 1971).

The international legal and policy framework relevant to mangrove ecosystems emerges from a patchwork of instruments which were not designed to function as a coherent whole, but rather address specific and separate objectives such as marine pollution control, biodiversity conservation, wetland protection or climate change (Slobodian et al. 2025; Slobodian and Badoz 2019). This structure is poorly suited to mangrove ecosystems and was not designed as a coherent system for the management of an ecosystem that spans land and sea, but instead developed through instruments with distinct mandates. Specifically, climate frameworks emphasise carbon sequestration (Paris Agreement 2015), while biodiversity schemes prioritise habitat conservation (CBD 2000), fisheries‐related instruments often emphasise fisheries productivity and nursery grounds (Hutchison et al. 2014) and coastal risk assessments value the storm protection given by mangrove forests (Krauss and Osland 2019).

The current instruments are rarely implemented in an integrated manner, and the governance fails to reflect the interconnected ecological functions of mangroves, yet do not provide comprehensive protection. A more coherent approach is needed to align existing instruments around shared conservation outcomes and there is a real need for a single coordinated legal strategy that connects existing categories and makes them mutually reinforcing.

This article analyses international mangrove governance through the lens of regime complex, ‘an array of partially overlapping and non‐hierarchical institutions governing a particular issue‐area’ (Raustiala and Victor 2004). This article focuses on four major multilateral environmental agreements that structure international governance relevant to mangrove ecosystems: UNCLOS, CBD, Ramsar and UNFCCC. These conventions each capture a different dimension of mangrove value and represent the principal international regimes through which mangroves are addressed, namely the law of the sea and marine protection, biodiversity conservation, wetland protection and climate mitigation. Regional agreements were excluded from the scope of the analysis with a view to examining fragmentation at the level of international governance.

The scope of this work is (i) to synthesise and present the principal international legal and policy frameworks relevant to mangrove conservation and restoration; (ii) to identify key limitations of the current legal frameworks; (iii) to propose practical recommendations to promote a more coherent global governance supported by ecological evidence.

Connecting international environmental law and coastal ecology is essential due to the current status of the world's mangroves. By bridging this gap, this study provides a practical defragmentation strategy and a roadmap for policymakers to align mangrove protection with binding climate and maritime obligations. This approach is directly relevant to governments, including those of coastal States updating their Nationally Determined Contributions (NDC), to the Conference of the Parties of the CBD and the Ramsar Convention, as well as to international courts and tribunals increasingly asked to interpret the environmental obligations under UNCLOS.

## 2 The Current Legal and Policy Framework Governing Mangroves

### 2.1 The United Nations Convention on the Law of the Sea

While governance of mangroves remains fragmented, the UNCLOS (1982) provides a binding global framework that can support more consistent protection at the land‐sea interface.

Pursuant to UNCLOS, States are under a general obligation to protect and preserve the marine environment. Article 192 establishes this obligation in broad terms, while Article 194 specifies that States must take all appropriate measures ‘to prevent, reduce and control pollution of the marine environment’ resulting from activities under their jurisdiction and control (UNCLOS 1982). These provisions apply to the marine environment as a whole, including coastal ecosystems located at the land‐sea interface, such as mangrove forests (Redgwell 2019).

Although framed in general language, the obligation set out in Article 192 has been interpreted as imposing concrete legal duties (Tanaka 2019). In *The South China Sea Arbitration (The Republic of Philippines v. The People's Republic of China)*, the Tribunal held that Article 192 constitutes a well‐established obligation binding on States Parties, the content of which is informed by the other provisions of Part XII of UNCLOS as well as by other applicable rules of international law (Permanent Court of Arbitration 2016, paragraph 941). The Tribunal further clarified that ‘protection’ concerns preventing future damage, whereas ‘preservation’ concerns maintaining or improving the present condition of the marine environment (Permanent Court of Arbitration 2016). Article 192 therefore entails a positive obligation to take active measures to protect and preserve the marine environment, and by implication a negative obligation not to degrade it (UNCLOS 1982).

The scope of this general obligation is further informed by the broader corpus of international environmental law. In particular, States are required to ensure that activities within their jurisdiction or control do not cause environmental harm to other States or to areas beyond national jurisdiction (duty to prevent transboundary environmental harm) (ICJ 1996; United Nations 1992a). As recognised in *The South China Sea Arbitration*, States have the ‘duty to prevent, or at least mitigate, significant harm to the environment when pursuing large‐scale construction activities’, a duty of due diligence that directly informs the interpretation of Article 192 (Permanent Court of Arbitration 2016; UNCLOS 1982). The obligation to protect and preserve the marine environment is an obligation of conduct rather than result (ICJ 2010; ITLOS 2011), requiring States to take all appropriate and reasonable measures in light of the risks involved.

Article 194 must also be read in conjunction with the definition of pollution of the marine environment set out in Article 1 (1) (4), which includes ‘(…) the introduction by man (…) of substances or energy into the marine environment (…) which results or is likely to result in deleterious effects on marine life, hazard to human health, hindrance to marine activities (…)’ (UNCLOS 1982). On this basis, climate change and greenhouse gas emissions may fall within the concept of marine pollution as they contribute to ocean warming, sea‐level rise and ecosystem degradation (Redgwell 2019). From this perspective, conservation of ecosystems that mitigate or buffer such impacts, including mangroves, is directly relevant to the fulfilment of States' obligations to protect and preserve the marine environment.

Beyond pollution control, Articles 192 and 194 also encompass obligations relating to the conservation of marine ecosystems and biodiversity (Roland Holst 2023). The International Tribunal for the Law of the Sea confirmed in the *Southern Tuna* cases that ‘the conservation of the living resources of the sea is an element in the protection and preservation of the marine environment’ (ITLOS 1999, paragraph 70). UNCLOS does not, however, make any explicit reference to mangrove ecosystems (UNCLOS 1982). This absence is unsurprising given the Convention's broad and framework character, and its reliance on coastal State implementation through sectoral regulatory approaches (Churchill and Lowe 1999). In practice, mangrove ecosystem protection therefore largely depends on how coastal States give effect to their UNCLOS obligations within areas under national jurisdiction, including the territorial sea and the Exclusive Economic Zone.

Finally, UNCLOS explicitly anticipates interaction with other international environmental regimes (UNCLOS 1982). Article 237 provides that the provisions of Part XII are without prejudice to the obligations assumed by States under other special conventions and agreements concerning the protection and preservation of the marine environment (UNCLOS 1982). This incorporation clause reflects the role of UNCLOS as an overarching framework. However, the openness of UNCLOS to other regimes does not necessarily imply that those regimes impose stronger or more comprehensive obligations. Some authors have argued that, while UNCLOS does not operate as a specialised instrument for mangrove protection, it provides a binding legal framework that imposes comparatively strong environmental obligations capable of including the protection of mangrove ecosystems and sets the foundation for the application of more specific international regimes (Roberts 2024; Boyle 2020).

As a universal, broad and potentially the most binding instrument, UNCLOS could function as an overarching framework within the mangrove regime complex. However, its general provisions and the discretion it leaves to coastal States mean that more specific instruments are required to fully address mangrove protection in practice.

### 2.2 The Convention on Biological Diversity

While UNCLOS can operate as a general backbone for marine environmental protection, other instruments more directly address biodiversity conservation and ecosystem‐based management.

The CBD establishes a global legally binding framework for the conservation of biodiversity, the sustainable use of its components, and the fair and equitable sharing of the benefits arising out of the utilisation of genetic resources (CBD 1992, Article 1). The CBD follows an ecosystem approach, which is a strategy for integrated management of land, water and living resources (CBD 2000) and explicitly integrates coastal and maritime ecosystems (e.g., operational objective 2.1 of the CBD Thematic Programme on the Conservation and Sustainable Use of Marine and Coastal Biological Diversity, CBD 1998). Mangroves fall within the scope of the CBD, which recognises them as ecosystems of high biological value (CBD 2004). Article 8(d) encourages the protection of ecosystems and natural habitats, while Article 8(f) promotes the rehabilitation and restoration of degraded ecosystems (CBD 1992). Despite this, the obligations under the CBD are often formulated in general terms and depend largely on national implementation, leading to inconsistent and fragmented application, and there is no harmonisation between the commitments and policies of States. Within the regime complex, the CBD, which is concerned with biodiversity conservation, does not interact with UNCLOS and there is no formal coordination between their respective Conferences of the Parties. It is interesting to note, however, that the CBD's ecosystem approach has led it to incorporate more climate‐related language than other multilateral environmental agreements (Elsler et al. 2022).

### 2.3 The Ramsar Convention on Wetlands

The Convention on the Conservation and Wise Use of Wetlands (Ramsar 1971) provides one of the most explicit international legal bases for the protection of mangroves (Sarker et al. 2025). Mangrove forests fall within the Convention's definition of wetlands and numerous designated Ramsar sites include mangrove ecosystems, such as the Parc national des Mangroves in Congo, the Amazon Estuary in Brazil and the Mangroves of Ambaro Bay in Madagascar (Ramsar 2026). The establishment of the International Mangrove Centre following COP 14 demonstrates increasing recognition of the importance of mangroves, including in the fight against climate change (Danone Fund for Nature 2010). However, the potential of the Convention, as the most explicit international legal basis for mangrove protection (Sarker et al. 2025) within the global mangrove regime complex, is limited by its largely non‐prescriptive approach. The Convention relies on voluntary site designation and does not specify binding legal protection requirements at the national level (Gaget et al. 2020; Bowman 2010); instead, Contracting Parties remain free to decide how designated sites are protected under national law (Ramsar 1971, Article 2, paragraph 6).

### 2.4 The UNFCCC and the Climate Regime

Climate governance provides an additional and increasingly influential lens for mangrove protection, particularly through the role of coastal ecosystems in mitigation, adaptation and climate resilience.

Under the UNFCCC (1992), the Conference of the Parties (COP) was established as the supreme body to review its implementation (Article 7). Emerging from this regime, the Kyoto Protocol is the only internationally legally binding instrument that introduced quantified greenhouse gas limitation and reduction (Kyoto Protocol 1997). Annex I countries have legally binding commitments to fix greenhouse gas emissions at a set percentage of base year emissions. However, the Kyoto Protocol did not develop a comprehensive approach to blue carbon ecosystems and coastal wetlands such as mangrove forests were not accounted for.

The Paris Agreement of 2015 (COP 21) marked an evolution (Paris Agreement 2015). Article 5 provides that Parties are encouraged to ‘conserve and enhance’ sinks and reservoirs of greenhouse gas (Paris Agreement 2015). Mangroves fall within this framework as efficient carbon sinks (Zhu and Yan 2022), and Parties are invited to write such measures in their NDC. However, NDCs remain nationally determined, non‐uniform and largely non‐binding. The Paris Agreement establishes binding procedural obligations to prepare, communicate and update NDCs, but it does not impose uniform, legally enforceable mitigation outcomes, which leads to uneven incorporation of coastal wetlands across countries.

Although the climate regime encourages parties to conserve sinks and reservoirs of greenhouse gases, mangrove ecosystems are not protected in their own right, but primarily as a means of climate mitigation. If it may encourage the conservation of mangroves as carbon sinks, this approach contrasts with that of the other instruments where the protection of the ecosystem itself constitutes the object of the legal obligation and could fail to address the obligations under the CBD and UNCLOS. These four conventions thus operate with distinct mandates and no formal coordination mechanism, leading to the governance fragmentation that this article seeks to address (Table 1).

**TABLE 1:** The four conventions (i.e., UNCLOS, CBD, Ramsar Convention and UNFCCC) presented in this study with related objectives, relevance to mangroves and limitations.

Convention	Primary objective	Relevance to mangroves	Limitations
UNCLOS (1982)	Issues relating to the law of the sea	Binding obligations to protect and preserve the marine environment, including coastal ecosystems (Articles 192 and 194)	No explicit reference to mangroves Due diligence so obligation of conduct, not result
CBD (1992)	Biodiversity conservation and sustainable use of its components	Mangroves as ecosystems of high biological value that parties are encouraged to protect	General obligations, non‐binding effect Implementation depends on national action plan
Ramsar Convention (1971)	Conservation and wise use of wetlands	Wetlands, mangrove ecosystems as designated Ramsar sites	Largely non‐prescriptive: relies on voluntary site designation
UNFCCC (1992)	Climate change mitigation and adaptation	Parties are encouraged to ‘conserve and enhance’ sinks and reservoirs of greenhouse gases	Mangroves addressed as carbon assets, conservation serves climates objectives

## 3 Discussion

### 3.1 Building a Solid Protection With Existing Instruments

Despite growing scientific awareness of the role that mangroves play in climate change mitigation and adaptation, they still have a marginal role in the international climate regime. This marginalisation is particularly evident when considering both the high vulnerability of many coastal States to climate impacts and the remarkable contribution that mangroves can offer to climate solutions (the Blue Carbon Initiative 2023; IPCC 2022). Furthermore, the benefits of natural disaster risk reduction (Sunkur et al. 2023), food security (Lukman et al. 2025), and the filtration of land‐based pollutants (Cappa et al. 2023) must be integrated to reflect the ecosystem's true multifunctional value.

UNCLOS, the CBD, the Ramsar Convention and the UNFCCC form a patchwork of overlapping yet uncoordinated legal instruments. Each addresses specific aspects of mangrove ecosystems, but none effectively captures their integrated ecological functions. This fragmentation undermines effective and sufficient protection and the policy and legal framework falls short of reflecting contemporary scientific understanding.

If the drivers of mangrove loss may vary across Southeast Asia for example, from aquaculture expansion in Indonesia and the Philippines to the rapid expansion of rice agriculture in Myanmar (Richards and Friess 2016), the absence of any binding international mechanism that requires States to subordinate these economic activities to their mangrove‐related conservation commitments remains a problem. Indonesia illustrates the consequences of this governance fragmentation. Following decentralisation reforms, mangrove management was transferred to regional authorities and distributed across five national ministries holding overlapping and often conflicting mandates without synchronised intersectoral regulations (Mursyid et al. 2021; Negara and Hutchinson 2021). Competing economic priorities, particularly mangrove conversion to aquaculture and oil palm plantations, have been identified as the principal drivers of mangrove loss in Indonesia (Richards and Friess 2016). None of the four international conventions discussed translated into effective protection in practice; UNCLOS obligations were not operationalised, CBD commitments were not uniformly implemented, Ramsar protection applied only to designated sites and the UNFCCC engaged mangroves primarily as carbon assets rather than addressing the institutional conditions that enable their clearance.

It is precisely because of the different services that mangroves provide that the legal and policy framework governing them is fragmented, but it is also precisely why it should not be. Rather than advocating for the creation of a new international instrument, stronger protection of mangroves can be achieved through an integrated interpretation and implementation of existing legal frameworks and institutional cooperation, guided by the best available scientific knowledge.

### 3.2 Integrating Mangrove Into Climate Commitments

Coastal States should systematically integrate mangrove conservation into their NDCs under the Paris Agreement. Such integration is consistent with Articles 4 and 5 of the Agreement, which respectively encourage mitigation measures and the conservation and enhancement of sinks and reservoirs of greenhouse gases. Mangrove forests, as highly efficient carbon sinks, clearly fall within this legal framework (Zhu and Yan 2022).

The Paris Agreement also establishes a five‐year cycle for communicating successive NDCs (Paris Agreement 2015), yet the current round of resubmission has progressed unevenly (UNFCCC 2025), and not all States have submitted updated contributions. A keyword screening—using the search term ‘mangrove’ and relevant translations—of NDC submissions indicates that explicit and detailed treatment of mangrove protection and restoration remains inconsistent across Parties (UNFCCC 2026).

Advocacy for the inclusion of mangroves in NDCs rests on three interrelated objectives: climate mitigation through carbon sequestration, adaptation through the strengthening of coastal resilience and biodiversity conservation. However, caution is required to ensure that mangrove integration is not driven solely by the economic appeal of blue carbon markets. Practical guidance is increasingly available to support science‐based, measurable and socially robust mangrove targets within NDCs (e.g., Wetlands International 2025); measures should be environmentally and ecologically appropriate, with safeguards to avoid ineffective restoration and to prevent marginalisation of local communities.

### 3.3 Operationalising Obligations Under UNCLOS

Under UNCLOS, States have the binding obligation to reduce or control pollution, including greenhouse gas emissions (Boyle 2020; UNCLOS 1982). Insofar as mangroves contribute to mitigating such pollution through carbon sequestration, their conservation and restoration could therefore be regarded as measures inherent to the implementation of Articles 192 and 194 of UNCLOS (UNCLOS 1982). This interpretation reflects (Gambardella 2018) a twofold understanding of the law of the sea in international environmental law as the regulation of States' ‘acceptable use of the ocean’ and, in parallel with the climate regime, the use of the ocean to offset the effects of human activities, despite the risks this poses to the marine environment. Agenda 21 reinforces this approach by emphasising the integrated coastal zone management and recognising the marine and coastal environment as an interconnected whole essential to sustainable development (United Nations 1992b).

Implementing these obligations in the context of mangrove ecosystems can be supported by pragmatic tools such as integrated coastal zone management, environmental and coastal development impact assessments, a deep understanding of land‐based sources of pollution, as well as monitoring systems that track the extent of mangrove ecosystems and ecological conditions rather than focusing solely on replanting activities (United Nations 1992b). In this light, UNCLOS would be better aligned to help transform mangrove action from a discretionary ‘good practice’ to a coherent set of due diligence measures that can be aligned and reinforced by biodiversity and climate commitments.

### 3.4 Scientific Consideration for Effective Policy Enforcement

Legal tools are a necessary first step, but scientific evidence is clear on the ecological priorities that policies must address. Continued clearing for aquaculture, agriculture and urban needs is a direct factor in the collapse of mangrove health and ecosystem services (Murray et al. 2020; Sasmito et al. 2019). Preventing loss by stopping deforestation remains the most reliable way to safeguard mangrove forests and the ecosystem services they provide. In this context, restoration is a valuable alternative but must remain a tool, not an equivalent alternative to protection of intact mangrove forests (Zimmer et al. 2022). Mangrove replanting can support forest recovery when it is ecologically justified, scientifically supported and implemented in the right sites (Lovelock et al. 2022; Lewis 2005), while being carefully designed, however it should not be used to legitimate further loss of intact mangrove forests. It is known that replanting in unsuitable areas such as mudflats or areas that were not historically mangrove forest, despite being cheaper, can lead to negative consequences for mangrove establishment (Lovelock et al. 2022; Lee et al. 2019). While the periodisation should be to allow natural regeneration where possible and avoid planting in unsuitable habitats (Beeston et al. 2023), there is also a growing consensus on the importance of the science‐based restoration done in the right place at the right time (e.g., Lewis 2005). Effective restoration must be guided by local hydrology, geomorphology, species‐site matching, as well as through active community interaction (which ensure an effective restoration and monitoring after restoration is completed) rather than setting targets solely for numerical purposes (Dalimunthe et al. 2025; Lewis 2005).

Greater protection of remaining intact mangrove areas is therefore needed, with a focus on effective management and policy enforcement. Conservation strategies must also move beyond a static view of mangrove distribution, as climate change may redistribute suitable mangrove habitats and alter the conditions required for their long‐term persistence (Ward et al. 2016; Cavanaugh et al. 2014). In addition to climatic pressures, local disease outbreaks and emerging health‐related stressors should also be considered in management plans, particularly where environmental changes may increase vulnerability (Nguyen et al. 2021; Osorio et al. 2016).

Mangrove survival also depends on land‐sea connectivity, requiring adequate freshwater supply, nutrients and sediment dynamics (Krauss et al. 2014). Upstream dams, water diversion, and altered watershed management can compromise mangrove forest health and recovery even where coastal protection exists (Friess et al. 2020; Lovelock et al. 2015). For this reason, integrating river basin processes with coastal management is essential to achieve long‐term conservation outcomes.

Finally, economic feasibility must be addressed alongside ecological priorities (Global Mangrove Alliance 2023). Low‐income countries often depend heavily on mangrove ecosystems, yet conservation capacity and funding may be limited and economic development pressures intense (Menéndez et al. 2024; Global Mangrove Alliance 2023). Science‐based conservation should therefore be paired with sustainable economic pathways that reduce dependency and offer alternatives to mangrove forest conversion for logging. In low‐income countries in which mangroves are a primary source of livelihood, a revision of laws and policies relating to mangroves may also be necessary. A governance framework that takes into account this fact, by recognising community management and encouraging sustainable use, is more likely to reduce illegal mangrove cutting and removal than conservation obligations alone (e.g., Datta et al. 2012).

## 4 Conclusion

Mangroves must be integrated into coastal planning and management as multifunctional systems that simultaneously support climate change mitigation, natural disaster risk reduction, fisheries, biodiversity conservation and pollution control. This article has examined the four main international conventions relevant to mangrove governance and identified a structural fragmentation that fails to provide sufficiently coherent legal protection. Together, these instruments constitute a regime complex that addresses mangroves through partially overlapping mandates but with insufficient coordination. The scientific evidence is now sufficiently robust to guide coordinated action, and the remaining challenge is to ensure that governance tools translate these priorities into coherent actions. Defragmenting the current framework does not require a new international convention, but making its instrument mutually reinforcing would help translate shared conservation outcomes into practical measures. This must be guided by the best available science, specifically to prioritise the prevention of further loss of intact mangrove forests, to ensure that restoration efforts are ecologically appropriate and that conservation obligations are paired with sustainable livelihood pathways, particularly in low‐income countries. To translate these socio‐ecological priorities into lasting protection, future research should assess how NDC commitments translate into enforceable domestic protection and examine how UNCLOS due diligence standards are interpreted by domestic courts in the context of coastal ecosystem management. In doing so, mangrove conservation can offer a highly effective pathway to advance multiple sustainable development goals, linking ecological integrity to climate resilience, food security and human well‐being.

## Author Contributions

**Marie Lorber:** conceptualization (equal), investigation (equal), visualization (equal), writing – original draft (equal), writing – review and editing (equal). **Paolo Cappa:** investigation (equal), visualization (equal), writing – original draft (equal), writing – review and editing (equal).

## Conflicts of Interest

The authors declare no conflicts of interest.

## Data Availability

No data were used in this study.
